# Angiogenic markers in difficult-to-treat psoriatic arthritis

**DOI:** 10.3389/fimmu.2026.1873191

**Published:** 2026-08-07

**Authors:** Amir Haddad, Elina Simanovich, Dunia Araide, Nili Stein, Noa Hayat, Katerina Milman, Tal Gazitt, Joy Feld, Muna Elias, Michal A. Rahat, Devy Zisman

**Affiliations:** 1 Department of Rheumatology, Carmel Medical Center, Haifa, Israel; 2 Ruth and Bruce Rappaport Faculty of Medicine, Technion-Israel Institute of Technology, Haifa, Israel; 3 Immunotherapy Laboratory, Carmel Medical Center, Haifa, Israel; 4 Department of Epidemiology, Clalit Health Services, Haifa, Israel

**Keywords:** angiogenic markers, biologic therapies, CD147 (EMMPRIN), difficult to treat (DTT), psoriatic arthritis (PsA)

## Abstract

**Background:**

Difficult-to-treat (DTT) psoriatic arthritis (PsA) is characterized by persistent disease activity despite treatment with multiple advanced therapies. Angiogenesis and extracellular matrix remodeling are key processes in chronic inflammatory arthritis, with CD147 acting as an important regulator.

**Objective:**

Our aim was to evaluate the circulating pro- and anti-angiogenic mediators and inflammatory cytokines in patients with DTT PsA compared with non-DTT PsA controls.

**Methods and results:**

Serum samples from 22 patients with DTT PsA were compared with matched samples from 25 non-DTT PsA patients who were on biologics or targeted therapies (non-DTT Bio) and with 25 non-DTT PsA patients who were naive to biologics and targeted therapies and received conventional disease-modifying antirheumatic drugs (cDMARDs) or non-steroidal anti-inflammatory drugs (NSAIDs) (non-DTT cDMARDs). The concentrations of angiogenic factors and inflammatory cytokines were determined with ELISA. Compared with both control groups, patients with DTT PsA demonstrated significantly higher serum levels of CD147 and increased activities of MMP-9 and proteasome 20S, which were measured by the cleavage of their respective fluorescent substrates. The levels of vascular endothelial growth factor (VEGF), TIMP-1, endostatin, and thrombospondin-1 (Tsp-1) did not differ significantly. The scratch assay with diluted serum samples, which was used to measure the ability of endothelial cells to migrate and close the gap, revealed enhanced angiogenic potential in the DTT group relative to the two control groups. In contrast, no differences between the groups were revealed in the serum levels of IL-17, IL-23, IL-22, IL-6, TNF-α, and TGF-β, and only IL-12p70 was reduced in the DTT relative to the control groups. The CD147 levels increased over time exclusively in the DTT PsA group.

**Conclusions:**

An enhanced angiogenic profile, highlighted by an elevated CD147, was found in patients with DTT PsA compared with non-DTT patients, requiring further studies to better understand its role in the pathogenesis of and as a biomarker in PsA.

## 1 Introduction

Psoriatic arthritis (PsA) is a chronic immune-mediated inflammatory disease characterized by musculoskeletal inflammation, enthesitis, dactylitis, and skin psoriasis (1). Despite significant therapeutic advances with biologic and targeted synthetic disease-modifying antirheumatic drugs (bDMARDs and tsDMARDs, respectively), a subset of patients continues to exhibit persistent disease activity despite multiple therapeutic interventions. This clinical state has been defined as difficult-to-treat (DTT) PsA, which is typically characterized by failure of at least two advanced therapies with different mechanisms of action (2).

The mechanisms underlying treatment resistance in PsA remain incompletely understood. Increasing evidence suggests that angiogenesis and inflammatory pathways are tightly interconnected in chronic inflammatory diseases (3, 4), as synovial angiogenesis contributes to leukocyte trafficking, pannus formation, and sustained inflammation within the joint microenvironment (5).

Several mediators regulate the balance between the pro- and anti-angiogenic signals in inflammatory tissues. The extracellular matrix metalloproteinase inducer (CD147/EMMPRIN) is a transmembrane glycoprotein that plays a central role in the regulation of matrix remodeling and angiogenesis (6), as well as in metabolic reprogramming and immune modulation (7). CD147 induces the expression of matrix metalloproteinases (MMPs), including MMP-9, and enhances the production of vascular endothelial growth factor (VEGF) (8). In addition, we have previously described how CD147 regulates the anti-angiogenic factor endostatin, a fragment derived from collagen XVIII that inhibits endothelial cell proliferation and angiogenesis (9), through the collaborative activity of MMP-9 and secreted proteasome 20S (10). Thus, CD147 is responsible for the balance between pro- and anti-angiogenic factors and the activation of the “angiogenic switch.”

Previous studies have demonstrated increased expression of CD147 and MMPs in inflammatory arthritis and psoriasis (4, 11), suggesting that these molecules may contribute to tissue remodeling and disease persistence. However, their role in DTT PsA has not been well characterized.

In this study, we looked for differences in the serum levels of key pro-angiogenic (CD147, VEGF, MMP-9, and TIMP-1) and anti-angiogenic [endostatin and thrombospondin-1 (Tsp-1)] mediators and the key pro- and anti-inflammatory cytokines associated with PsA to identify potential mechanisms and biomarkers underlying the refractory disease course in DTT patients.

## 2 Methods

### 2.1 Study population

Patients attending a combined rheumatology–dermatology clinic with special interest in PsA were offered to participate in a clinical database. Patients were enrolled consecutively during routine visits at different stages of their disease course rather than at the time of diagnosis or at treatment initiation. The patients in this well-characterized cohort were followed according to a standard protocol that includes a detailed history, physical examination, use of medication, and laboratory and radiographic assessments. All individuals fulfilled the Classification Criteria for Psoriatic Arthritis (CASPAR) and were eligible for inclusion (*N* = 328). Of this cohort, only 22 patients were classified as having difficult-to-treat (DTT) PsA, i.e., with persistent active disease defined as being in a non-MDA (minimal disease activity) state (12) despite treatment with at least two bDMARDs or tsDMARDs with different mechanisms of action.

Each DTT patient was matched with at least one non-DTT PsA patient of similar age and disease duration. Non-DTT patients were further stratified into two groups: patients who received biologics or targeted therapies (non-DTT Bio) and patients who were naive to biologics or targeted synthetic therapies (bDMARDs/tsDMARDs) and received conventional disease-modifying antirheumatic drugs (cDMARDs) or non-steroidal anti-inflammatory drugs (NSAIDs) only. Because patients were enrolled in the database at different stages of their disease, a standardized baseline serum sample was not available for all patients. For each patient, the clinical visit demonstrating the highest recorded articular and skin disease activity in the database was selected for analysis.

The clinical assessment documented the presence of tender joints (of the 68 assessed joints), swollen joint (total 66 joints and clinically damaged joints (of 68 assessed joints). The Visual Analogue Scale for pain, the Patient and Physician Global Assessment, and the Disease Activity in Psoriatic Arthritis (DAPSA) composite score (13) were evaluated in all patients at each time point. Enthesitis was assessed using the Spondyloarthritis Research Consortium of Canada Enthesitis Index (14), and dactylitis was assessed using the Leads Dactylitis Index (15). Skin assessment included the Psoriasis Area and Severity Index (PASI) and body surface area. Nail involvement was also recorded. The Health Assessment Questionnaire (HAQ) patient-reported outcome was collected to assess physical functioning. The demographics, disease characteristics, and comorbidities including fibromyalgia were recorded. Serum samples were collected and stored according to standardized protocols.

### 2.2 Sandwich enzyme-linked immunosorbent assay

The concentrations of the pro- and anti-angiogenic factors CD147, MMP-9, VEGF, endostatin, Tsp-1, and TIMP-1 and the cytokines IL-17A, IL-23, IL-22, IL-12p70, IL-6, TNF-α, and TGF-β were evaluated using the human DuoSet ELISA kits (R&D Systems, Minneapolis, MN, USA) according to the manufacturer’s instructions. Serum samples were diluted in dilution buffer [1% bovine serum albumin (BSA) in phosphate-buffered saline (PBS)] according to previous calibration experiments. For all pro-angiogenic factors, sera were diluted 1:100, except for endostatin (1:20), VEGF (1:5), and Tsp-1 (1:1,000). For all cytokines, sera were diluted 1:5, except for IL-22 (1:50).

### 2.3 MMP-9 activity

ELISA plates were coated with a capture antibody that specifically recognizes MMP-9 (2 mg/ml; R&D Systems) to differentiate between MMP-9 activity and MMP-2 activity, which is also capable of breaking down the fluorescent substrate. After overnight incubation and a wash with PBS, 15 μl of different serum samples was added to the wells and incubated for 1 h, following three more washes with PBS. Subsequently, the MMP-9 captured onto the plate was incubated with 120 μl of the MMP-9 fluorescent substrate DNP-PLGMWSR (50 mM; Cayman, Ann Arbor, MI, USA) in assay buffer (25 mM tricine, 0.2 M NaCl, 10 mM CaCl_2_, and 0.05% Brij-35) for 5 min. The supernatants were transferred to a black 96-well plate, and the fluorescence that resulted from the cleavage of the substrate was read at 490 nm excitation and 520 nm emission. Recombinant MMP-9 that was activated overnight with aminophenylmercuric acetate (APMA, 1 mM; Merck/Sigma-Aldrich, Darmstadt, Germany) was used as a positive control.

### 2.4 Proteasome 20S activity

In a black 96-well plate, 15 μl of different serum samples was mixed with 120 μl of the proteasome 20S assay buffer (0.5 M EDTA, 1 M HEPES buffer, and 1 M DTT) and the proteasome 20S fluorescent substrate SUC-LLVY-2R110 (6 µM; AAT Bioquest, Sunnyvale, CA, USA) for 5 min. The fluorescence emitted from the cleaved peptide was read at 490 nm excitation and 520 nm emission. The proteasome 20S preparation that was activated for 1 h with 0.5% sodium dodecyl sulfate (SDS) served as a positive control.

### 2.5 Scratch assay

We used the scratch assay to assess the angiogenic potential of the factors accumulated in the serum samples, as described previously (4). Briefly, the human endothelial cell line EaHy926 (ATCC CRL-2922) was grown overnight to confluency in full medium [10% fetal calf serum (FCS), 1% penicillin–streptomycin antibiotics, 1% glutamine, 1% amphotericin B, and 2% HAT (hypoxanthine, aminopterin, and thymidine)] in Dulbecco’s modified Eagle’s medium (DMEM). All reagents were from IMBH-Import and Marketing, Beit HaEmek, Israel. A scratch was made to the confluent layer using a sterile tip, with the detached cells washed away with PBS. Afterward, the different serum samples were diluted 1:2 with the medium, and 300 μl, in the absence or presence of the anti-CD147 antibody (h161-pAb, 2 ng/ml) or its control rabbit IgG (2 ng/ml), was applied onto the endothelial cells. The cells were incubated for 18 h, and images of the scratch were acquired immediately after the scratch was applied (0 h) or at the end of the incubation (18 h). The area of the gap at 18 h was measured using the wound healing size tool plugin (16) for ImageJ (version 1.54q; National Institute of Health, Bethesda, MD, USA) and subtracted from the area at 0 h to determine the average migration area.

### 2.6 Statistical analysis

Continuous variables were expressed as the mean ± standard error (SEM). Comparisons among the three study groups were performed using the non-parametric Kruskal–Wallis ANOVA test, followed by Dunn’s multiple comparison test. Pairwise comparisons between groups were adjusted using Bonferroni correction to account for multiple testing, which was also applied to the comparison of angiogenic and cytokine levels. Statistical significance was defined as *p* < 0.05. Based on the observed sample sizes, assuming 80% power and a two-sided alpha level of 0.025 for pairwise comparisons (DDT *vs*. non-DTT Bio and DDT *vs*. non-DTT cDMARDs), the study was sufficiently powered to detect a large effect size (Cohen’s *d* above 1).

## 3 Results

A total of 22 patients with DTT PsA were matched with 25 non-DTT Bio patients and 25 biologic-naive non-DTT patients receiving DMARDs or NSAIDs (non-DTT cDMARDs). The mean age of the cohort was 62.3 ± 15.5 years, with a mean disease duration of 19.4 ± 11.1 years. The baseline demographic characteristics were comparable across the three groups, with no significant differences in sex distribution, age at psoriasis or PsA diagnosis, body mass index, ethnicity, smoking status, alcohol use, or the majority of the comorbidities depicted in **Table 1**. The DTT PsA patients and the non-DTT Bio patients demonstrated significantly high disease burden compared with those in the non-DTT cDMARDs group. Specifically, these two groups had higher tender joint (TJ) and swollen joint (SJ) counts compared with the biologic-naive non-DTT group. The DAPSA scores were high in all groups, with the highest levels observed in the DTT group. However, there was no statistical difference between these two groups in terms of the SJ counts or DAPSA scores, and only the enthesitis scores were higher in the DTT group. Functional disability, as measured by the HAQ, was also significantly worse in the DTT group. In addition, fibromyalgia was observed in only 3 of the 22 patients in the DTT group. However, the presence of tenosynovitis and dactylitis was more prevalent in non-DTT patients. Patient-reported outcomes, including global assessment and pain scores, showed a trend toward worse disease in the DTT group, although these did not reach statistical significance. As expected, the use of advanced therapies (anti-TNF, anti-IL-17, anti-IL-12/23 agents) was significantly higher in the DTT group, reflecting their refractory disease course. No significant differences were observed between groups in the PASI scores, presence of nail lesions, and the body surface areas affected with psoriasis.

**Table 1:** Baseline demographic, clinical, and treatment characteristics of the patient groups.

Variable	DTT PsA (*n* = 22)	Non-DTT Bio (*n* = 25)	Non-DTT cDMARDs (*n* = 25)	*p*-value
Sex, male, *n* (%)	8 (36.4)	10 (40.0)	9 (36.0)	0.950
Age at diagnosis of PsA	43.9 ± 17.6	41.6 ± 11.5	43.8 ± 14.7	0.993
Age diagnosis of psoriasis	32.3 ± 18.0	33.6 ± 16.3	33.4 ± 16.4	0.914
Ethnicity, Jews, *n* (%)	21 (95.5)	21 (84.0)	22 (88.0)	0.574
Body mass index	*n* = 22 29.2 ± 5.9	*n* = 24 31.0 ± 9.6	*n* = 22 29.88 ± 5.9	0.962
Smoking, *n* (%)				0.315
Never	11 (50.0)	16 (64.0)	16 (64.0)	
Past	4 (18.2)	5 (20.0)	7 (28.0)	
Current	7 (31.8)	4 (16.0)	2 (8.0)	
Alcohol, *n* (%)	11 (50.0)	13 (52.0)	11 (44.0)	0.842
Disability	19 (86.4)	23 (100)	23 (100)	0.031
Cardiac disease, *n* (%)	4 (18.2)	9 (36.0)	6 (24.0)	0.363
Ischemic heart disease, *n* (%)	1 (4.5)	1 (4.0)	3 (12.0)	0.611
Diabetes, *n* (%)	4 (18.2)	5 (20.0)	5 (20.0)	>0.99
HTN, *n* (%)	4 (18.2)	8 (34.8)	5 (21.7)	0.397
Inflammatory bowel disease, *n* (%)	1 (4.5)	0	0	0.310
Liver disease, *n* (%)	6 (27.3)	5 (21.7)	2 (8.7)	0.301
Uveitis	2 (9.1)	0	0	0.09
Inflammatory spinal pain, *n* (%)	5 (22.7)	2 (8.0)	6 (24.0)	0.298
Tender joint count, Mean ± SD Median (IQR)	9.0 ± 7.5 7 (4–11)	5.8 ± 4.4 7 (1.5–9)	3.7 ± 3.2 2 (1–6)	0.010^b^
Swollen joint count Mean ± SD Median (IQR)	5.5 ± 3.5 7(2–7.3)	5.2 ± 3.8 5 (1–8)	2.7 ± 3.4 1 (0–4)	0.009^b^^,^ ^c^
DAPSA score	28 ± 13.1	24.9 ± 15.1	16.8 ± 9.6	0.007^b^
SPARCC Enthesitis Index Mean ± SD Median (IQR)	2.0 ± 1.9 2 (0–3.3)	0.8 ± 1.2 0 (0–1.5)	0.88 ± 1.4 0 (0–1)	0.026^a^
Active dactylitis, *n* (%)	0	3 (12)	1 (4.0)	0.317
Active tenosynovitis, *n* (%)	3 (13.6)	3 (12.0)	10 (41.7)	0.007
Damaged joint count (at least 1), *n* (%)	3 (13.6)	7 (28.0)	3 (12.5)	0.324
Fibromyalgia, *n* (%)	3 (13.6)	0	0	0.027
PASI Mean ± SD Median (IQR)	2.5 ± 3.8 1.3 (0–3.8)	1.3 ± 1.4 1.2 (0–2.2)	2.8 ± 3.4 1.8 (0.83–3.4)	0.145
Psoriasis body surface area, median (IQR)	1 (0–2)	1 (0–2)	1 (1–4)	0.368
Presence of nail lesions, *n* (%)	8 (36.4)	6 (24.0)	8 (33.3)	0.628
Patient global assessment of PsA Mean ± SD Median (IQR)	6.5 ± 2.5 7 (5.8–8)	5.3 ± 2.6 6 (3–7)	6.3 ± 7.7 5 (1.5–8)	0.194
Patient global assessment of arthritis Mean ± SD Median (IQR)	6.8 ± 2.7 7 (6–9)	5.4 ± 2.7 6 (3–7.5)	4.9 ± 3.3 5 (2–8)	0.085
Physician global assessment Mean ± SD Median (IQR)	4.0 ± 1.7 4 (2–5)	3.2 ± 1.8 3 (2–4)	3.3 ± 2.1 3 (1.3–4.8)	0.228
Pain VAS (Visual Analogue Scale) Mean ± SD Median (IQR)	6.3 ± 2.8 7 (5–9)	5.9 ± 2.9 7 (3–8)	4.9 ± 2.8 5 (3–7.5)	0.215
Health assessment questionnaire (HAQ) Mean ± SD Median (IQR)	1.5 ± 0.76 1.7 (0.94–2.0)	1.1 ± 0.71 1.3 (0.5–1.6)	0.73 ± 0.76 0.5 (0.13–1.4)	0.005^b^
Treatment ever				
Anti-TNF, *n* (%)	21 (95.5)	22 (88.0)	0	<0.001^b^^,^ ^c^
Anti-IL-17, *n* (%)	19 (86.4)	4 (16.0)	0	<0.001^a^^,^ ^b^
Anti-IL-12, *n* (%)	10 (45.5)	1 (4.0)	0	<0.001^a^^,^ ^b^
Anti-IL-23, *n* (%)	9 (40.9)	0	0	<0.001^a^^,^ ^b^
JAK inhibitors, *n* (%)	4 (18.2)	2 (8.0)	0	0.072

*DTT*, difficult-to-treat [psoriatic arthritis patients; non-DTT Bio, PsA patients receiving biologics or targeted therapies; non-DTT cDMARDs, PsA patients who never received biologics and are treated with conventional disease-modifying antirheumatic drugs (cDMARDs) or non-steroidal anti-inflammatory drugs (NSAIDs)]; *DAPSA*, Disease Activity in Psoriatic Arthritis; *SPARCC*, Spondyloarthritis Research Consortium of Canada; *PASI*, Psoriasis Activity Score Index.

^a^ Comparison between the DTT and non-DTT Bio groups after Bonferroni correction.

^b^ Comparison between the DTT and non-DTT cDMARDs groups after Bonferroni correction.

^c^ Comparison between the non-DTT Bio and non-DTT cDMARDs groups after Bonferroni correction.

**Figure 1:**
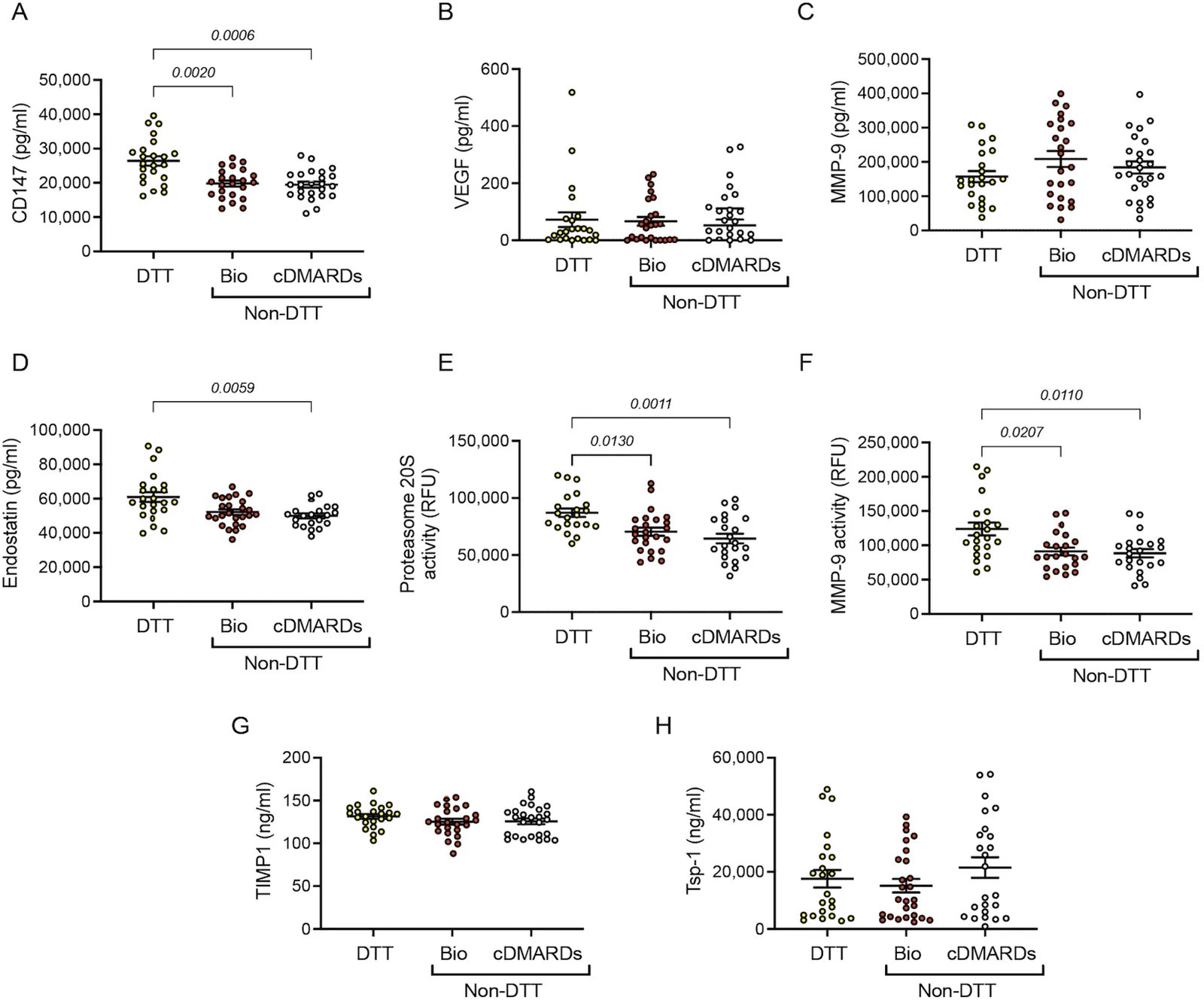
Serum concentrations of the pro- and anti-angiogenic factors in the study groups. **(A–D)** Serum levels of CD147 **(A)**, vascular endothelial growth factor (VEGF) **(B)**, MMP-9 **(C)**, and endostatin **(D)**. **(E)** Proteasome 20S activity. **(F)** MMP-9 activity. **(G, H)** Serum levels of TIMP-1 **(G)** and Tsp-1 **(H)**. Yellow circles denote the DTT group, red circles the non-difficult-to-treat (DTT) Bio group, and open circles the non-DTT conventional disease-modifying antirheumatic drugs (cDMARDs) group. Data are presented as the mean ± standard error and analyzed using the non-parametric Kruskal–Wallis ANOVA test followed by Dunn’s multiple comparison test.

Firstly, we evaluated the serum levels of the pro- and anti-angiogenic factors. The levels of CD147 were significantly elevated by approximately 1.35-fold in DTT patients (26,410 ± 1,379 pg/ml) compared with both the non-DTT biologic-experienced (19,493 ± 885 pg/ml, *p* < 0.0007) and the non-DTT biologic-naive patients (19,774 ± 857 pg/ml, *p* < 0.0012), as shown in **Figure 1A**. In contrast, both the levels of VEGF (**Figure 1B**) and MMP-9 (**Figure 1C**) were not different between the three groups. However, the enzymatic activity of MMP-9, which was determined by its ability to specifically cleave its fluorescently labeled substrate, was significantly enhanced by approximately 1.37-fold in the DTT group (123,861 ± 9,446 relative fluorescence units (RFU)] relative to the non-DTT Bio group (90,067 ± 5,620 RFU, *p* < 0.0131) and the non-DTT cDMARDs group (89,328 ± 6,258 RFU, *p* < 0.0177) (**Figure 1F**). Interestingly, the levels of the anti-angiogenic factor endostatin were also enhanced by approximately 1.2-fold in the DTT group (60,951 ± 2,832 pg/ml) relative to the non-DTT Bio group (52,079 ± 1,573 pg/ml, *p* < 0.0487) and the non-DTT cDMARDs group (50,105 ± 1,410 pg/ml, *p* < 0.0071) (**Figure 1D**), whereas the levels of TIMP-1 and Tsp-1 (**Figures 1G, H**) were unchanged. The activity levels of the secreted proteasome 20S (**Figure 1E**) were also enhanced by approximately 1.3-fold in the DTT group (87,123 ± 3,690 RFU) relative to the control non-DTT Bio group (68,314 ± 3,285 RFU, *p* < 0.0043) and the control non-DTT cDMARDs group (66,774 ± 4,574 RFU, *p* < 0.0041). These results are in accordance with our previous studies (10, 17).

**Figure 2:**
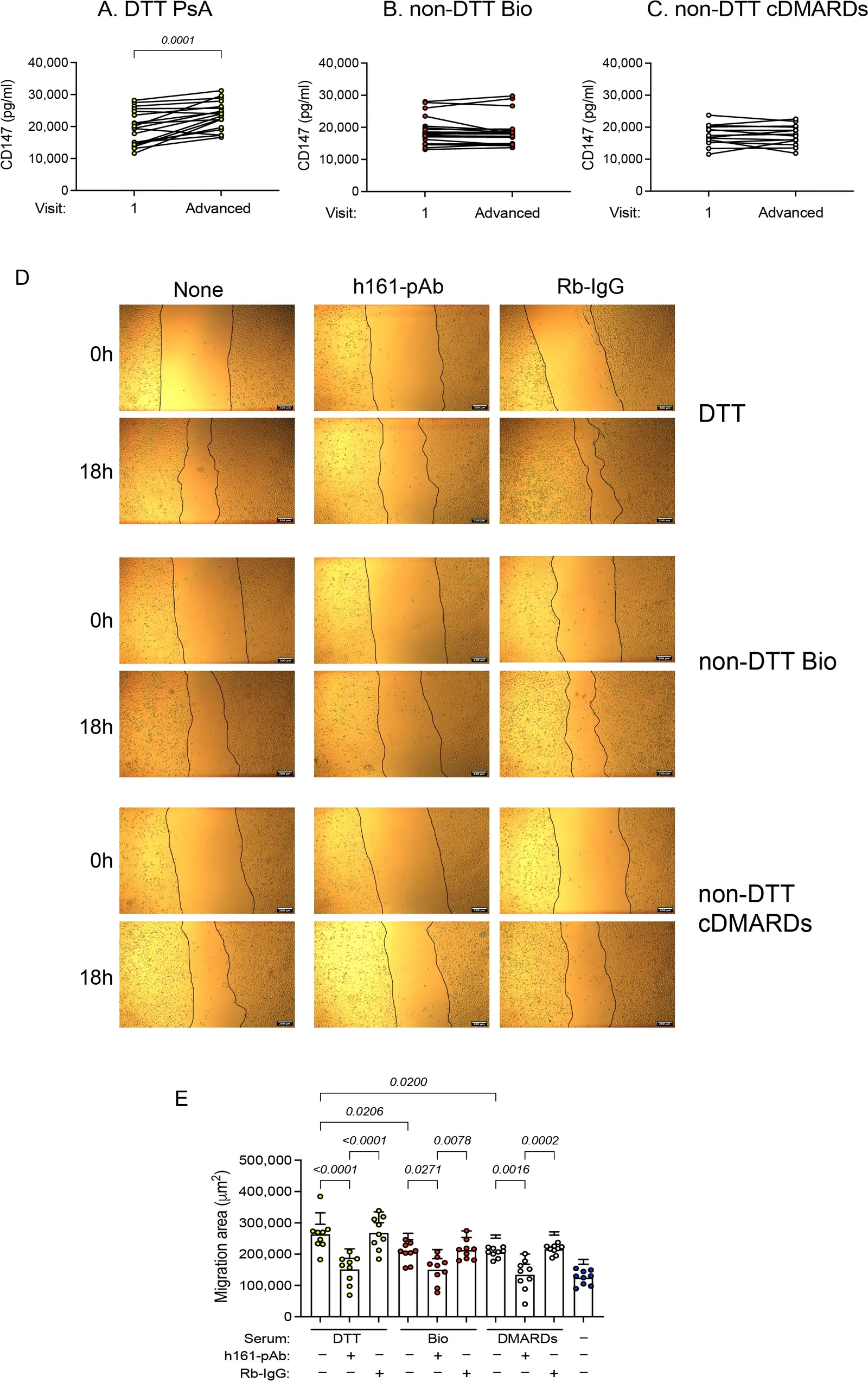
The serum CD147 levels changed over time only in the difficult-to-treat (DTT) group, and the angiogenic potential is higher. Serum CD147 was measured for each patient in the baseline visit and in the most active visit in all groups, and the change per patient was determined. **(A)** DTT group (*n* = 19). **(B)** Bio group (*n* = 21). **(C)** Disease-modifying antirheumatic drugs (DMARDs)/non-steroidal anti-inflammatory drugs (NSAIDs) group (*n* = 14). Data were analyzed using the two-tailed paired *t*-test. Serum samples from the three groups were diluted and applied onto a scratched endothelial layer in the absence or presence of the anti-CD147 antibody (h161-pAb, 2 ng/ml) or the control antibody (rabbit-IgG, 2 ng/ml), as described in *Section 2*. **(D, E)** Representative images **(D)** and calculation of the area the cells migrated to **(E)**. Yellow circles indicate the DTT group, red circles the non-DTT Bio group, and open circles the non-DTT cDMARDs group. Blue circles indicate no addition of serum, describing the basal migration rate of endothelial cells. In all groups, addition of h161-pAb reduced the migration rate back to the basal levels, suggesting that CD147 is a regulator of the angiogenic potential.

To further explore the potential role of CD147 in treatment resistance in the DTT group, we examined longitudinal changes in the serum CD147 levels between the most active visit and the baseline visit, when the baseline sample was available. A significant increase of approximately 1.2-fold in the CD147 levels over time was observed exclusively in the DTT PsA group (*p* = 0.0001) (**Figure 2A**). In contrast, no significant change was detected in the non-DTT biologic-experienced group (**Figure 2B**) or in the biologic-naive patients treated with conventional DMARDs (**Figure 2C**).

To ascertain that the differences observed in the serum levels of the pro- and anti-angiogenic factors for the DTT PsA group relative to the two control groups also represent potential angiogenic activity, we next examined these serum samples in the functional wound healing assay. To establish whether CD147 is the cause of this activity, we added the control rabbit IgG antibody or the neutralizing anti-CD147 antibody previously developed in our laboratory to the samples before applying them onto the scratch endothelial layer. Similar to the differences in the serum concentrations of CD147, the migration of the endothelial cells was enhanced in the DTT group by approximately 1.25-fold relative to the non-DTT Bio group and the non-DTT cDMARDs group (**Figures 2D, E**). The addition of the neutralizing h161-pAb that inhibits CD147 markedly reduced the migration across the three groups (by 1.7-, 1.4-, and 1.5-fold in the DTT, non-DTT Bio, and non-DTT cDMARDS groups, respectively), bringing the migration back to the baseline levels without any addition of serum or antibodies, while the addition of the control rabbit IgG antibody had no significant effect (**Figures 2D, E**).

**Figure 3:**
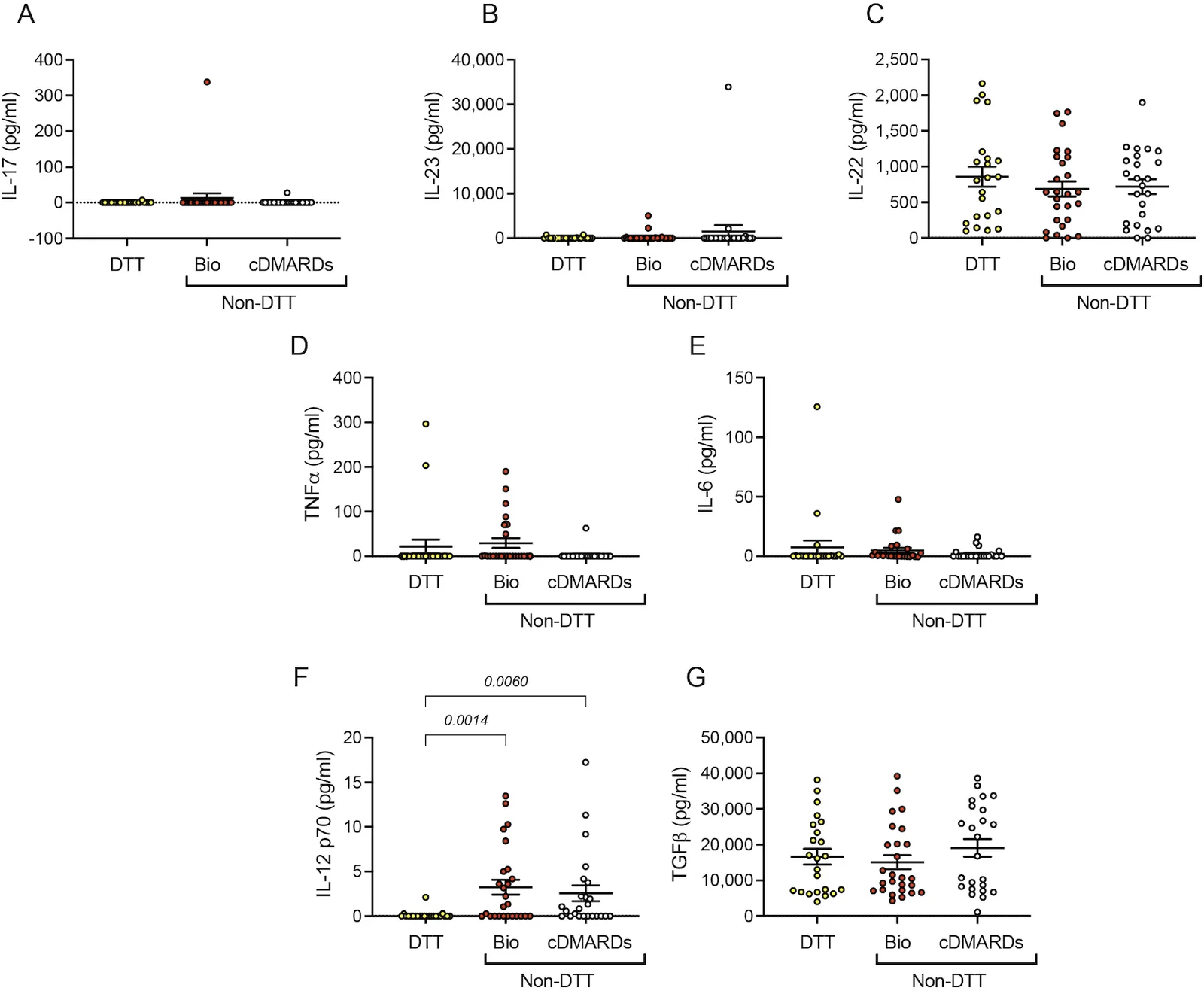
Serum concentrations of the pro- and anti-inflammatory cytokines in the study groups. Serum levels of IL-17A **(A)**, IL-23 **(B)**, IL-22 **(C)**, TNF-α **(D)**, IL-6 **(E)**, IL-12p70 **(F)**, and TGF-β **(G)**. *Yellow circles* denote the difficult-to-treat (DTT) group, red circles the non-DTT Bio group, and open circles the non-DTT conventional disease-modifying antirheumatic drugs (cDMARDs) group. Data are presented as the mean ± standard error and analyzed using the non-parametric Kruskal–Wallis ANOVA test followed by Dunn’s multiple comparison test.

As pro-inflammatory cytokines have been implicated in the pathogenesis of PsA, we next examined differences in the levels of some of these cytokines between the three groups. Surprisingly, circulating cytokine levels of IL-17A, IL-23, IL-22, TNF-α, IL-6, and TGF-β did not reveal any significant differences between the DTT group and the control groups (**Figure 3**). However, the IL-12p70 levels were significantly reduced by approximately 25-fold in DTT patients (0.115 ± 0.09 pg/ml) relative to the non-DTT Bio group (3.13 ± 0.87 pg/ml, *p* < 0.0054) and the non-DTT cDMARDs group (2.69 ± 0.84 pg/ml, *p* < 0.0014), suggesting a potential immune involvement that merits further investigation, especially as these concentration were not significantly influenced by treatment with either IL-12/23 or IL-23 inhibitors.

## 4 Discussion

In this study, we asked why some patients with active PsA are more difficult to treat than others. To this end, we measured the levels of several angiogenic and inflammatory cytokines in serum samples obtained from patients defined as having DTT PsA, a population that remains poorly characterized despite increasing clinical recognition (18), and compared them with those in patients receiving treatment with biologics, mainly inhibitors of the TNF-α or IL-17 pathways, and in non-DTT patients who were biologic-naive. Notably, although the DTT group exhibited a high disease burden, the active joint counts and the psoriasis severity were comparable to those of the biologic-experienced non-DTT group, suggesting that these groups may be clinically difficult to distinguish based on their active joint counts and DAPSA scores. Interestingly, active tenosynovitis was more prevalent in the non-DTT biologic group. This finding may reflect referral or treatment selection bias within our tertiary care cohort or suggest differences in the clinical phenotypes between treatment groups. Further studies are needed to determine the underlying explanation.

Our findings demonstrate that patients with DTT PsA are associated with elevated serum levels of CD147, although not statistically significant, and an increase in the activity of MMP-9 and proteasome 20S, as well as higher levels of endostatin, although these did not reach significance. CD147 and MMP-9 are considered pro-angiogenic factors, whereas endostatin is a potent anti-angiogenic factor; the concomitant elevation of these factors seems paradoxical. However, we have previously described similar findings in the serum of patients with rheumatoid arthritis (RA) (10, 17) and demonstrated that CD147 acts as an allosteric cofactor for secreted proteasome 20S and MMP-9, enhancing their cooperative activities that are responsible for cleaving collagen XVIIIA to produce endostatin (2, 5). Therefore, the increase in CD147 and MMP-9 activity, which was higher than the increase in endostatin (~1.35-fold *vs*. 1.2-fold) may suggest that the balance between the pro- and anti-angiogenic factors favors the former and may tilt the balance toward activating the angiogenic switch. This is also supported by the results of the scratch assay, which showed that the overall mediators in the serum samples added to the endothelial layer promoted, rather than inhibited, enhanced angiogenic potential over the natural migration of endothelial cells without any addition. Moreover, the involvement of MMP-9 in matrix remodeling may implicate this process, in addition to the increased angiogenic potential, in the resistance to treatments and refractory inflammation as it may facilitate the recruitment of different immune cells. Taking into account the inhibitory effect of the anti-CD147 antibody (h161-pAb) on the migration of endothelial cells, which is greater in the DTT group compared with the two control groups, CD147 emerges as an important regulator of the angiogenic switch, as was demonstrated previously (19), which is associated with disease refractoriness.

A particularly notable finding of our study is the longitudinal increase in CD147 levels observed only in the patients in the DTT group. This further supports the role of CD147 in enhancing angiogenesis, which might contribute to the increased resistance to treatment and in the pathophysiology of refractory PsA. Moreover, it is possible that elevated levels of CD147 may serve as a potential biomarker for identifying patients at risk of developing a DTT disease course, a possibility that requires further investigation. However, it should be noted that, relative to that in healthy individuals, the CD147 level is also elevated in patients with RA and PsA who do not develop a refractory disease, as we have previously shown (4, 20). Therefore, further studies involving a large cohort of patients followed up over time are needed to determine the range of CD147 concentrations that indicate inflammation or a refractory disease.

Of note is that although enhanced angiogenic potential was functionally demonstrated, the levels of classical angiogenic mediators, such as VEGF, did not differ between groups. These findings point to the complexity of the angiogenic process and suggest that angiogenesis is regulated through multiple pathways and mediators beyond VEGF signaling, supporting previous reports indicating that CD147 can promote angiogenesis in multiple VEGF-independent mechanisms (21). We found no clear differences in the levels of the cytokines that are clearly associated with PsA, such as IL-17A, IL-23, TNF-α, and IL-6, in the DTT group, challenging the idea that refractory disease is simply “more inflammation.” However, this should be interpreted with caution as we did not measure tissue-level concentrations, which may be more relevant than serum levels. Bearing in mind that CD147 also has an inflammatory role, particularly in the regulation of macrophages (22) and T regulatory cell (Treg) activation (10, 17), the elevation in its levels over time in the DTT group only may point to influences on these cell types that drive an immunosuppressive response, which we have not yet investigated. Supporting this premise is a study demonstrating that, in mice where CD147 was silenced and were infected with *Pseudomonas aeruginosa*, the Th1-type cytokines (e.g., IFN-γ, IL-12, and IL-18) were significantly upregulated, whereas the Th2-type cytokines (e.g., IL-4, IL-5, and IL-10) were downregulated (23). The cytokines we chose to measure do not necessarily point to the activation of immunosuppressive cell types, such as M2-activated macrophages and Tregs. However, the downregulation of IL-12, which was not confounded by treatment background, may suggest that the pro-inflammatory response is kept in check in these DTT patients. Thus, a more comprehensive study assessing the concentrations of cytokines that characterize immunosuppressive responses, as well as the isolation of monocytes and T cells from these patient groups, may reveal whether these cells have a role in mediating the refractory disease. Overall, our findings support the idea that refractory disease may be driven not by increased systemic inflammation but rather by alternative mechanisms that may include enhanced angiogenesis, tissue remodeling, and perhaps even increased immunosuppressive response.

Several limitations should be acknowledged. The sample size was relatively small, and the cross-sectional design precludes assessment of causality. In addition, serum biomarkers may not fully reflect the local synovial microenvironment. Treatment heterogeneity is also an inherent limitation of our study, as patients were receiving different therapeutic regimens at the time of serum sampling. To explore whether treatment exposure influenced circulating CD147 levels, we compared the CD147 concentrations across the different treatment categories and found no significant differences between treatment groups, suggesting that the observed differences in CD147 are unlikely to be primarily driven by the specific therapeutic regimen. Nevertheless, this study provides novel insights into angiogenic dysregulation in DTT PsA and points to CD147 and IL-12 as potential biomarkers of disease refractoriness.

To conclude, patients with DTT PsA demonstrate elevated CD147 levels along with elevated proteasome 20S and MMP-9 activity. These findings suggest that a dysregulated angiogenic environment, which may increase over time in DTT PsA, is mediated by CD147, highlighting this protein as a cause of the enhanced angiogenic potential and continued matrix remodeling in these patients. Further studies that will clarify the mechanistic role of CD147 in DTT patients are warranted and may contribute further to personalized therapeutic strategies.

## Data Availability

The raw data supporting the conclusions of this article will be made available by the authors, without undue reservation.
